# Effects of L-β-Galactoglucan Supplementation on Growth Performance, Palatability, and Intestinal Microbiota in Adult Beagle Dogs

**DOI:** 10.3390/metabo15030160

**Published:** 2025-02-28

**Authors:** Chenghe Chang, Zifeng Gu, Lingling Du, Jiantao Guo, Ying Yang, Zhenlong Wu

**Affiliations:** 1 State Key Laboratory of Animal Nutrition and Feeding, College of Animal Science and Technology, China Agricultural University, Beijing 100193, China; c15737958269@163.com (C.C.); 15717922003@163.com (Z.G.); wuzhenlong@cau.edu.cn (Z.W.); 2 Chengdu Sydix Biotech Co., Ltd., Chengdu 610000, China; lynn@sydix.com.cn; 3 Beijing Shanchongshuifu Technology Development Co., Beijing 100084, China; 18201546422@163.com

**Keywords:** L-β-galactoglucan, dog, growth performance, palatability, gut microbiota

## Abstract

**Background:** This study was conducted to investigate the effects of different levels of L-β-galactoglucan on growth performance, palatability, and health condition of dogs. **Methods:** A total of 32 healthy beagle dogs (2.0 ± 0.5 yr; 13.2 ± 2.1 kg) were randomly assigned into four treatment groups, with 8 dogs in each group. The dogs were fed basal diets supplemented with 0 (control), 0.25, 0.5, or 1% L-β-galactoglucan. **Results:** The results showed that the feed intake ratio of the dogs in the Low_Gal (0.25%) group was significantly higher (*p* < 0.05) as compared with the control (Con) group. The low-density lipoprotein cholesterol (LDL-C) levels of the Mid_Gal (0.5%) group showed a trend toward lower levels as compared with the control (Con) group (*p* = 0.069). Compared with the control (Con) group, the alpha diversity of the bacterial flora of the Shannon index of the Mid_Gal (0.5%) group was significantly higher (*p* < 0.05). The Simpson index was significantly reduced (*p* < 0.05), and a PCoA indicated a significant change in the gut microbiota structure among the four groups (*p* < 0.05). The relative abundance of *Blautia* and *Peptoclostridium* in the Low_Gal (0.25%) group was significantly higher as compared with the control (Con) group (*p* < 0.05). **Conclusions:** These results indicated that L-β-galactoglucan exhibited a positive effect on improving the palatability and gut microbiota of dogs.

## 1. Introduction

The gastrointestinal (GI) tract is now recognized as the largest endocrine organ in mammals, and its functionality is known to play a critical role in overall health and well-being [1,2]. Beyond its functions in digestion and nutrient absorption, GI health involves the complex interaction between gut microbiota, fermentation processes, and metabolite production [3]. Among these, the gut microbiota and its metabolites have emerged as important biomarkers of GI function across multiple species, including dogs [4,5,6].

Maintaining a balanced intestinal microbial community is essential for supporting immune function, nutrient absorption, and metabolic processes in dogs. An imbalance in the gut microbiota can either enhance or impair intestinal immunity [7]. Diet plays a pivotal role in shaping the gut microbiome and exerts a greater influence than environmental factors [8]. Prebiotics, such as lactulose, fructooligosaccharides, inulin, β-glucans, yeast cell walls, and mannan oligosaccharides, are known to have beneficial effects on the gut microbiome and are commonly used in the pet food industry [9]. Research has shown that oyster polysaccharides exert significant regulatory effects on the abundance of gut microbiota [10]. Zhang et al. demonstrated that oligosaccharide supplementation modulates canine gut microbiota and leads to the production of fermentation metabolites (e.g., short-chain fatty acids such as acetate and butyrate), indicating their potential value as dietary supplements to improve canine digestive health [11]. Hokkyo et al. found that continuous ingestion of galacto-oligosaccharides could positively modulate the gut microbiota of household dogs, supporting their role in promoting microbial homeostasis and enhancing canine health [12]. Given the promising findings from these studies, it is of significance to identify new and safe prebiotics to enhance gastrointestinal health in dogs. L-β-galactoglucan, a highly branched, high-molecular-weight polysaccharide composed of β-D-glucose and β-D-galactose, was recently isolated from agrobacterium FN01. Despite the functional role of polysaccharides derived from plants, fungi, and bacteria in regulating gut flora [13], it remains unknown whether L-β-galactoglucan, an agrobacterium-derived polysaccharide, can modulate intestinal microbiota and, therefore, contribute to the intestinal health of dogs. This study was conducted to evaluate the effects of L-β-galactoglucan on various health parameters in dogs, including growth performance, nutrient digestibility, palatability, hematology, serum biochemistry, fecal quality, and gut microbiota composition, with the aim to provide insights into how L-β-galactoglucan promotes gastrointestinal balance and the overall well-being of dogs, therefore contributing to advancements in pet health and nutrition.

## 2. Materials and Methods

### 2.1. Animals and Diets of the Feeding Experiment

A total of 32 healthy adult male beagle dogs (2.0 ± 0.5 years; 13.2 ± 2.1 kg) were randomly assigned into four treatment groups, with 8 dogs per group (*n* = 8/group). Each beagle dog was kept in an individual cage (1.0 × 1.0 m), with constant temperature (25 ± 1 °C) and humidity (50 ± 10%), under a 12 h light–dark cycle (light from 06:00 to 18:00). This study was conducted at the Experimental Animal Centre of Beijing Shanchongshuifu. The dogs were fed 400 g of dried food twice daily (at 09:00 and 17:00) to meet their energy requirements, calculated approximately according to the standard of the National Research Council (NRC, 2006). The ingredients and nutritional levels of the diet are presented in Table 1. The control (Con) group received a basal diet without any additional prebiotics to ensure that observed effects were solely attributable to L-β-galactoglucan supplementation. L-β-galactoglucan was added to the diets of the beagle dogs at a percentage of 0.25% (Low_Gal group), 0.5% (Mid_Gal group), or 1% (High_Gal group) of the basal diet, which was provided by Chengdu Sydix Biotech Co., Ltd. (Chengdu, China). The experimental duration lasted for 28 days to allow for a comprehensive assessment of the dietary interventions.

### 2.2. Palatability Trial

A two-day, two-bowl palatability test was conducted with 30 dogs. Following the international standard for palatability comparison, each trial was conducted over two days, with the position of the bowls switched on the second day. The control (Con) group was compared with the Low_Gal (0.25% L-β-galactoglucan) group, and the Mid_Gal (0.5% L-β-galactoglucan) group was compared with the High_Gal (1% L-β-galactoglucan) group. During each trial, two stainless steel bowls, each containing 400 g of the respective diet, were presented to the dogs for 30 min. Dietary preference was evaluated by calculating the intake ratio between the two diets and by recording the first choice [14]. The intake ratio, a measure of dietary preference, was calculated using the following formula:

Intake ratio (diet A/B) = Intake (diet A/B)/Total intake (diet A + diet B)

### 2.3. Growth Performance

All the dogs were weighed individually on the 1st day and the 28th day of the trial to record their initial body weight and final body weight.

### 2.4. Sample Collection

Fecal scores were evaluated throughout the entire experimental period following Carciofi’s method [15]: 1 = watery, liquid that can be poured; 2 = soft and unformed, taking the shape of the container; 3 = soft, formed, moist, and retains its shape; 4 = hard, formed, dry, and firm yet soft; 5 = hard, dry pellets, small and firm. Fresh fecal samples were collected from the metabolic cages of each dog within 15 min of defecation, transferred to sterile 5 mL fecal collection tubes, and then stored at –80 °C for gut microbiota analyses. During the last 3 days of the experiment, fecal samples were collected and stored at −20 °C for analysis of apparent total tract digestibility (ATTD). At the end of the experimental period, 5 mL of blood was collected via jugular venipuncture from each dog before the morning feeding to ensure standardized conditions across all groups. The blood samples were divided into serum and hematology components using a serum separator and Ethylene Diamine Tetraacetic Acid tubes, respectively. After centrifugation at 3000× *g* for 15 min at 4 °C, the serum was stored at −20 °C for subsequent analysis. Concurrently, hair from each dog’s back was collected and scored from 1 to 3 based on its brightness, softness, and skin condition. The hair quality score was the sum of these three scores.

### 2.5. Measurement of Apparent Total Tract Digestibility (ATTD) of Nutrients

The fecal and feed samples were thawed and then dried in a forced-air oven at 65 °C for 72 h (Shanghai Huyueming Scientific Instrument Co., Ltd., Shanghai, China). The ash, crude protein (CP), and ether extract (EE) of the fecal samples were analyzed using AOAC methods 942.05, 990.03, and 996.01, respectively. The content of crude fiber (CF) was determined using F57 filter bags and fiber analyzer equipment (Fiber Analyzer; Ankom Technology, Macedon, NY, USA) according to the method described in [16]. The acid-insoluble ash (AIA) in both the feed and fecal samples was measured as described by the Standards Press of China (2009). The ATTD of nutrients was calculated using the following formula:

ATTD (%) = 100 − [100 × (b × c)/(a × d)]

where a = the content of a nutrient in the feed, b = the content of a nutrient in the feces, c = the AIA content in the feed, and d = the AIA content in the feces.

### 2.6. Blood and Serum Biochemical Analysis

Hematological analyses were performed using an automated analyzer to determine the complete blood count, encompassing white blood cells (WBCs), red blood cells (RBCs), hemoglobin (HGB), hematocrit (HCT), mean cell volume (MCV), platelets (PLTs), and mean corpuscular hemoglobin (MCH). Serum biochemical profiles were evaluated for total protein (TP), glucose (GLU), albumin (ALB), globulin (GLB), aspartate aminotransferase (AST), alanine aminotransferase (ALT), triglycerides (TGs), high-density lipoprotein cholesterol (HDL-C), and low-density lipoprotein cholesterol (LDL-C), utilizing a Roche cobas 6000 c501 analyzer (Roche Diagnostics Co., Ltd., Mannheim, Germany).

The serum concentrations of immunoglobulins, specifically immunoglobulin A (IgA), immunoglobulin M (IgM), and immunoglobulin G (IgG), were quantified using enzyme-linked immunosorbent assay (ELISA) kits (MEIMIAN, Jiangsu Meimian Industrial Co., Ltd., Jiangsu, China) and measured with a microplate reader (Molecular Devices, LLC, CA, USA).

### 2.7. Intestinal Microbiota Analysis

Fecal microbial DNA was extracted using the E.Z.N.A. Soil DNA Kit, and the genomic DNA was visualized through 1% agarose gel electrophoresis. Amplification and library construction were carried out using universal primers 341F (5′-CCTACGGGRSGCAGCAG-3′) and 806R (5′-GGACTACVVGGGTATCTAATC-3′). Next-generation sequencing was carried out on an Illumina HiSeq platform following the protocol provided by Majorbio Biopharm Technology Co., Ltd. (Shanghai, China). Sequencing reads were processed on the QIIME (Quantitative Insights Into Microbial Ecology) platform, following steps for low-quality sequence removal, filtering, chimera removal, and taxonomic classification. The sequences were clustered based on ≥97% similarity into operational taxonomic units (OTUs). All subsequent analyses of α-diversity (Chao1, ACE, Shannon, and Simpson) and β-diversity were performed using the normalized data output from QIIME2. Principal coordinate analysis (PCoA) was used to identify differences in bacterial communities between the groups. Linear discriminant analysis (LDA) effect size (LEfSe), T-tests, and Kruskal–Wallis rank sum tests were conducted to evaluate bacterial abundance differences among the groups.

### 2.8. Statistical Analysis

All graphs were generated using GraphPad Prism, version 8, and Adobe Illustrator 2024. The data were analyzed using SPSS 26.0 (SPSS, Inc., Chicago, IL, USA). The results in this experiment were analyzed by one-way analysis of variance (ANOVA). Differences between groups were assessed using Duncan’s multiple comparisons test. The data are presented as the mean ± SEM (Standard Error of the Mean). A significance level of *p* < 0.05 was considered statistically significant, while *p* > 0.05 was considered not statistically significant.

## 3. Results

### 3.1. Palatability

Different concentrations of L-β-galactoglucan were tested for palatability, and it was found that the feed intake rate of the dogs in the Low_Gal (0.25%) group was significantly higher as compared with the control (Con) group (*p* < 0.05), and there was no difference in feed intake between the two groups. As for the mid_Gal group and the High_Gal group, the feed intake showed an increasing trend (*p* = 0.083) between the Mid_Gal group and the High_Gal group, while there was no difference in the intake ratio between these two groups (Table 2).

### 3.2. Growth Performance, Fecal, and Hair Quality Assessment

As shown in Table 3, L-β-galactoglucan supplementation had no effect on the fecal score or the hair score as compared with the control (Con) group. Compared with the control (Con) group, L-β-galactoglucan treatment showed a statistically significant trend toward a reduction in body weight change (*p* < 0.05).

### 3.3. The Apparent Total Tract Digestibility (ATTD) of Nutrients

As shown in Table 4, the apparent total tract digestibility (ATTD) of crude protein, ether extract, organic matter, and crude fiber were determined. L-β-galactoglucan had no significant effect on the apparent digestibility of nutrients, including CP, EE, CF, and OM (*p* > 0.05).

### 3.4. Hematology

The results of the hematology analyses are presented in Table 5. As shown, L-β-galactoglucan supplementation had no effect on the number of WBCs, RBCs, HGB, PLT, HCT, MCV, or MCH as compared with the control (Con) group (*p* > 0.05). All hematology profiles remained within normal reference ranges throughout the trial.

### 3.5. Serum Biochemistry Parameters

As shown in Table 6, L-β-galactoglucan supplementation had no effect on blood glucose, total protein, albumin, or HDL-C levels as compared with the control (Con) group. The dogs in the Mid_Gal (0.5%) group showed a trend toward lower LDL-C levels as compared with the control (Con) group (*p* = 0.069). Compared with the control (Con) group, the Mid_Gal (0.5%) group exhibited a trend toward higher globulin levels (*p* = 0.052) and a trend toward lower triglyceride levels in the Mid_Gal (0.5%) and High_Gal (1%) groups (*p* = 0.06).

### 3.6. Immunoglobulin

As presented in Table 7, L-β-galactoglucan supplementation had no effect on the serum levels of IgA, IgM, or IgG as compared with the control (Con) group (*p* > 0.05).

### 3.7. Intestinal Microbiota

**Figure 1:**
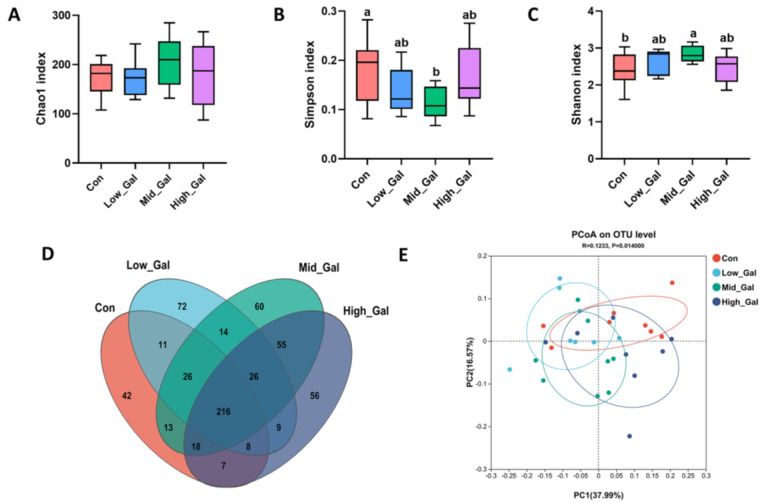
Effects of L-β-galactoglucan on the alpha diversity and beta diversity of the intestinal microbiota of dogs. (**A**) Chao index; (**B**) Simpson index; (**C**) Shannon index; (**D**) Venn diagram showing the OTUs of fecal microorganisms; (**E**) Plot of principal coordinate analysis. The beagle dogs were fed 0% (Con), 0.25% (Low_Gal), 0.5% (Mid_Gal), and 1% (High_Gal) L-β-galactoglucan. ^a b^ different letters above bars indicate statistically significant differences among groups (*p* < 0.05).

**Figure 2:**
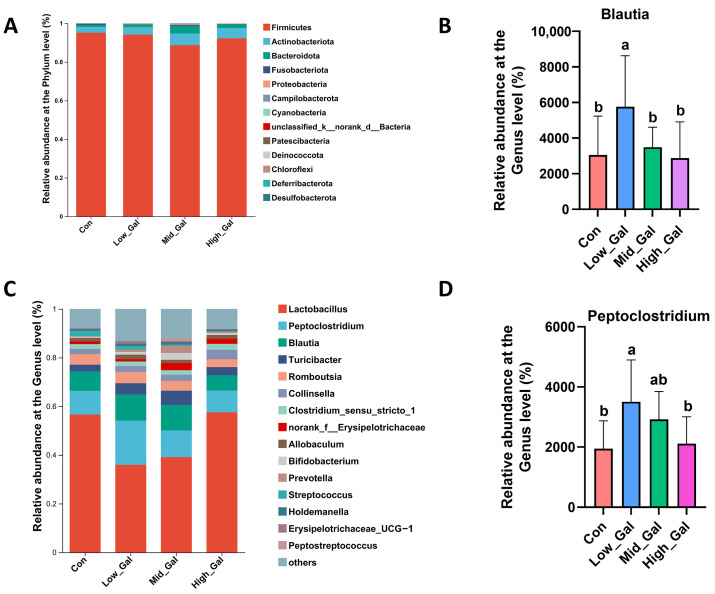
Effects of L-β-galactoglucan on the fecal microbial composition of dogs. (**A**) Phylum-level community composition. (**B**) The relative abundances of blautia at the genus level. (**C**) The relative abundances of bacteria at the genus level. (**D**) The relative abundances of peptoclostridium at the genus level. The beagle dogs were fed 0% (Con), 0.25% (Low_Gal), 0.5% (Mid_Gal), and 1% (High_Gal) L-β-galactoglucan. ^a b^ different letters above bars indicate statistically significant differences among groups (*p* < 0.05).

Alpha diversity, a metric of species richness and evenness within samples, was evaluated using the Chao1, Simpson, and Shannon indexes. The Shannon index of the flora in the Mid_Gal (0.5%) group was significantly increased (*p* < 0.05) as compared with the control (Con) group, accompanied by a corresponding reduction in the Simpson index (*p* < 0.05), but there was no significant difference in the Chao1 index among the four groups (Figure 1). Venn diagrams were employed to count the number of species that are shared and exclusive to multiple groups or samples. The Venn (Figure 1D) result showed that 216 OTUs were shared, and 42, 72, 60, and 56 OTUs were unique to the control (Con) group, Low_Gal (0.25%), Mid_Gal (0.5%), and High_Gal (1%) groups. Figure 1E illustrates the principal component analysis (PCoA) of fecal microorganisms, which shows that the composition of fecal microbiota was significantly changed among the four groups (*p* < 0.05). Figure 2A shows that Firmicutes made up the majority of the fecal microbiota at the phylum level, followed by Actinobacteriota, Bacteroidota, and Fusobacteria. L-β-galactoglucan supplementation had no effect on the relative abundance of Firmicutes, Actinobacteriota, Bacteroidota, or Fusobacteria as compared with the control (Con) group (*p* > 0.05). As illustrated in Figure 2C, the dominating bacteria at the genus level were *Lactobacillus*, *Peptoclostridium*, *Blautia*, *Turicibacter*, *Romboutsia*, *unclassified_f_Peptostreptococca*, and *Collinsella*. Of note, the relative abundance of *Blautia* was significantly higher in the Low_Gal (0.25%) group as compared with the control (Con) group (*p* < 0.05). Similarly, the relative abundance of *Peptoclostridium* was significantly higher in the Low_Gal group (0.25%) as compared with the control (Con) group (*p* < 0.05).

**Figure 3:**
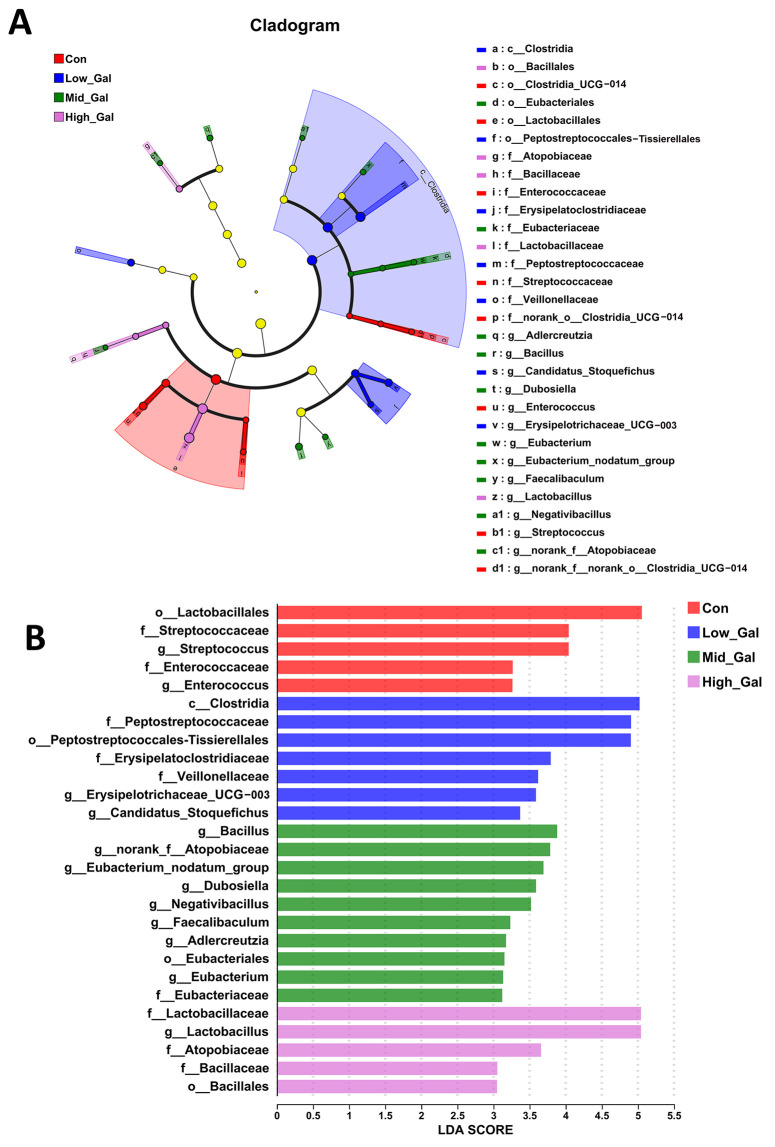
Different taxa microbe analyses of fecal contents based on the LEfSe method. (**A**) Cladogram of the microbe structure axis. The circles from the inside to the outside represent the classification level (phylum, class, order, family, and genus). (**B**) The default parameters were LDA score > 2 and *p* < 0.05. Different colored regions represent different constituents. The beagle dogs were fed 0% (Con), 0.25% (Low_Gal), 0.5% (Mid_Gal), and 1% (High_Gal) L-β-galactoglucan.

As shown in the cladogram of the microbe structure axis (Figure 3A), a significant change in microbes was found in the four groups. As can be seen in Figure 3B, the linear discriminant analysis (LDA) scores of 2.0 or higher were confirmed by the linear discriminant analysis effect size (LEfSe). The results showed that *Lactobacillales*, *Streptococcus*, *Streptococcaceae*, *Enterococcaceae*, *Enterococcus*, and *unclassified_p__Firmicutes* were predominant in the control (Con) group. For the Low_Gal (0.25%) group, *Clostridia*, *Peptostreptococcaceae*, and *Erysipelatoclostridiaceae* were the dominant species. For the Mid_Gal (0.5%) group, *Bacillus* and *norank_f__Atopobiaceae* were the dominant species. For the High_Gal (1%) group, *Lactobacillaceae* and *Lactobacillus* were the dominant species.

## 4. Discussion

This study investigated the effects of L-β-galactoglucan supplementation on growth performance, nutrient digestibility, palatability, hematology, serum biochemistry, fecal quality, and intestinal flora composition in beagle dogs. In recent decades, extensive research has explored the role of prebiotics in enhancing canine growth, immune function, and intestinal balance [17,18]. However, the specific impact of L-β-galactoglucan in dogs has not been previously documented, leaving a gap in the literature, which this study aimed to address. Our investigation focused on how L-β-galactoglucan influences palatability and overall canine health. The findings of this study demonstrate the positive effects of L-β-galactoglucan on both palatability and intestinal microbiota in dogs.

When introducing new components to pet food, it is crucial to ensure they do not negatively impact palatability. In this study, dogs in the Low_Gal (0.25%) group showed a significantly higher feed intake ratio as compared with the control (Con) group (*p* < 0.05), indicating a positive impact on dietary acceptability in dogs. The Mid_Gal (0.5%) group showed an increasing trend in feed intake as compared with the High_Gal (1%) group, indicating a dose-dependent effect on palatability within the tested range. These findings align with previous research, which has shown that the addition of modest amounts of prebiotic fibers can enhance the palatability of pet food [19,20]. It should be emphasized that several factors, including the intrinsic properties of ingredients, food processing techniques, physical structure, and the interaction between aroma, texture, and taste, contribute to the overall palatability of pet food [21]. Nevertheless, the two-day palatability assessment used in this study is limited, as it may not fully capture long-term dietary preferences or adaptation effects. Prolonged assessments, such as week-long trials, may prove to provide deeper insights into sustained dietary acceptance and better reflect real-world feeding scenarios. This limitation is recognized, and it is recommended that future studies incorporate longer evaluation periods to address this issue.

Fecal quality is often viewed by pet owners as a key indicator of gastrointestinal function and overall digestive health in dogs [17]. In this experiment, L-β-galactoglucan did not appear to affect fecal characteristics or metabolite concentrations. Canine health was assessed through parameters such as fecal mass, body weight, and nutrient digestibility, which are critical for nutrition and metabolism. Our results indicate that the inclusion of L-β-galactoglucan in the diet did not significantly impact the digestibility of nutrients, which is consistent with previous studies showing that prebiotics had no effect on nutrient digestibility in healthy dogs [18,21,22,23,24]. L-β-galactoglucan, as a prebiotic, is not digested in the small intestine; instead, it is mainly utilized by gut microbes. It makes sense that L-β-galactoglucan does not directly alter the apparent digestibility of other nutrients. However, a study reported a decrease in the apparent digestibility of dry matter, organic matter, and minerals in dogs supplemented with beta-glucan [25]. Conversely, the addition of dehydrated yeast cultures increases the apparent nutrient digestibility of crude fiber and decreases the digestibility of crude protein and nitrogen-free extract in dogs (*p* < 0.05) [26]. The discrepancies between our data and data from previous studies are currently unknown. The age, sex, life stage, and diet composition, as well as the capability of animals to utilize L-β-galactoglucan, might be involved in and contribute to discrepancies, as shown in previous studies [19,25]. In this experiment, the dogs in all the groups supplemented with L-β-galactoglucan showed a statistically significant trend toward a reduction in body weight as compared with the control (Con) group. Further studies are required to uncover the potential mechanisms responsible for this phenomenon.

Serum biochemical profiles and immunoglobulin levels provide essential insights into an animal’s overall health and immunological status. LDL-C is an apolipoprotein that primarily transports cholesterol from the liver to the rest of the body. In this experiment, LDL-C levels in the Mid_Gal (0.5%) group showed a trend toward lower levels as compared with the control group, indicating a potential effect of L-β-galactoglucan on lipid metabolism. Previous research has shown that dogs administered 1% oat β-glucan exhibited lower serum total cholesterol concentrations as compared with the control group, along with decreased low-density lipoprotein and very-low-density lipoprotein concentrations (*p* < 0.05) [25]. Zhou et al. [20] showed that Modified Highland Barley supplementation significantly reduced TC, TG, and LDL-C levels, while HDL-C levels showed an increasing trend in mice. A meta-analysis found that a daily intake of at least 3 g of oat beta-glucan reduced serum total cholesterol and LDL-C levels [27]. Additionally, mannan oligosaccharides combined with 1,3 β-glucan significantly reduced plasma cholesterol (*p* < 0.001) and LDL levels (*p* < 0.05) in hens [28]. These findings indicate a potential dose-dependent effect of L-β-galactoglucan on LDL-C levels. Alternatively, gut microorganisms may metabolize galactoglucan, enhancing gut microbial composition, which may affect hepatic lipid metabolism and decrease LDL-C synthesis. The trend toward lower triglyceride levels in the supplemented groups (*p* = 0.06) further reinforces the role of L-β-galactoglucan in lipid regulation, likely through its prebiotic effects and subsequent impact on hepatic metabolism.

Regarding immune function, the dogs in our study showed no significant changes in serum immunoglobulin levels (IgA, IgM, and IgG) regardless of the supplementation level. A previous study showed that β-glucan supplementation enhanced immunoglobulin levels in mice [29], dogs [30], and calves [31]. These findings indicate that L-β-galactoglucan is typically well-tolerated and does not adversely affect the immune system in dogs, although its effects on immunomodulation may vary by species or dosage.

The gut microbiota, which exists in symbiosis with the host, plays an important role in maintaining health [32]. Gut microbiota diversity is typically assessed using alpha and beta diversity metrics. Alpha diversity quantifies species richness and evenness within individual samples. In this experiment, the Shannon index was significantly higher in the Mid_Gal (0.5%) group as compared with the control group (*p* < 0.05), indicating an increase in species richness and a more complex and possibly healthier microbial ecosystem. Similarly, the Simpson index, which quantifies species evenness, was considerably lower in the Mid_Gal (0.5%) group. The decrease in the Simpson index suggests a more balanced microbial population, reducing the dominance of a few species and contributing to a stable and functional gut environment. Greater diversity and richness in the intestinal flora are typically associated with improved health outcomes [33], although the specific mechanisms and conditions under which this occurs require further investigation. For instance, the addition of Artemisia ordosica crude polysaccharide reduced the Simpson estimator in rumen fluid as compared with the control group (*p* < 0.05) [34]. Likewise, Schutte et al. [35] reported enhanced microbial diversity and positive metabolic effects after consuming high-fiber or whole-grain diets.

In healthy dogs, Firmicutes, Bacteroidetes, Proteobacteria, Fusobacteria, and Actinobacteria are recognized as the dominant phyla [36]. Several *Blautia* species have recently been shown to have beneficial effects on host function, such as suppressing *Vibrio cholera* colonization [37], improving inflammatory responses in a dextran sulfate sodium-induced colitis mouse model [38], ameliorating obesity and type-2 diabetes [39], and protecting against enteric viral infection [40]. *Peptoclostridium* species, particularly those within the Clostridium genus, have been identified as beneficial bacteria with significant roles in gut health and homeostasis. Research indicates that these bacteria can help modulate immune responses, reduce inflammation, and promote the production of short-chain fatty acids like butyrate, which are crucial for intestinal health. Furthermore, some studies indicate their potential as probiotics due to their ability to restore gut microbiota balance, especially following antibiotic treatment or gastrointestinal disturbances [41]. The results of this study also showed that L-β-galactoglucan did not alter the core composition of the normal flora of fecal microflora. However, at the genus level, the relative abundance of the beneficial bacterium *Blautia* was significantly higher in the Low_Gal group (0.25%) as compared with the control group (*p* < 0.05). Similarly, the relative abundance of the beneficial bacterium *Peptoclostridium* was significantly higher in the Low_Gal group (0.25%) as compared with the control group (*p* < 0.05). Previous studies have shown that β-glucan modulates microbiota composition, and supplementation with β-glucan derived from cereal or microbial sources increases the intestinal population of beneficial bacteria in both pigs [42] and chickens [43]. Santos et al. [26] reported an increase in the proportion of *Actinobacteria* and *Sterolactobacilli* and a decrease in *Fusobacteria* in response to the addition of dehydrated yeast cultures. However, the gut microbiota composition can vary significantly between individuals, which may lead to different responses to dietary interventions [44]. These findings indicate that L-β-galactoglucan may act as a fermentable substrate for specific gut microbes, leading to the production of SCFAs such as butyrate, which supports gut barrier integrity and modulates host immune responses.

To further validate the prebiotic effects of L-β-galactoglucan, we conducted a Linear Discriminant Analysis (LDA) Effect Size (LEfSe) analysis. This analysis revealed increased LDA scores for certain genera in response to L-β-galactoglucan supplementation, indicating that L-β-galactoglucan can modulate gut microbiota by promoting beneficial genera while possibly suppressing harmful ones.

The long-term effects of L-β-galactoglucan supplementation in canines merit further investigation. The data presented in the current and previous studies with polysaccharides indicated that the application of L-β-galactoglucan is unlikely to exert adverse effects; instead, it may provide benefits, including improved gut health, weight management, and immune function. However, the short duration of this study emphasizes the necessity for long-term research to validate its safety and efficacy in chronic dietary use.

## 5. Conclusions

In conclusion, the supplementation of 0.25 and 0.5% L-β-galactoglucan in the diets of beagle dogs can improve palatability and gut microbiota diversity. Furthermore, L-β-galactoglucan supplementation at the 0.5% level contributes to a reduction in LDL-C levels. L-β-galactoglucan supplementation also promotes the proliferation of probiotics, as the relative abundance of beneficial bacteria such as *Blautia* and *Peptoclostridium* was significantly increased in the Low_Gal (0.25%) treatment group. These results support the potential of L-β-galactoglucan as a functional ingredient for improving intestinal health and dietary acceptability in dogs.

## Figures and Tables

**Table 1:** Ingredients and chemical compositions of the experimental diets.

Ingredient (%)	Amount	Chemical Composition (%)	Content
Chicken meal	20.0	Dry matter	94.7
Duck meat meal	17.0	Crude protein	21.9
Corn meal	15.0	Ether extract	8.50
Potato meal	7.00	Ash	7.50
Soya bean meal	10.0	Crude fiber	2.60
Pea meal	7.00	Acid insoluble ash	0.90
Chicken oil	8.50	Phosphate	0.78
Soya bean oil	2.00	Gross energy, MJ/kg	19.1
Purified cellulose	2.00		
Chicken liver meal	2.00		
Canine flavoring agent	4.00		
Premix	2.00		

**Table 2:** Effect of L-β-galactoglucan on the palatability of the dogs.

Items	Con	Low_Gal	SEM	*p*-Value	Mid_Gal	High_Gal	SEM	*p*-Value
FI (g/d)	43.4	50.3	5.74	0.554	52.6	34.4	5.238	0.083
IR (%)	42.3 ^b^	57.7 ^a^	0.036	0.018	53.6	46.4	0.349	0.304
PR (%)	47.4%	52.6%			61.5%	38.5%		

^a,b^ means with different superscript letters were significantly different (*p* < 0.05). FI = feed intake; IR = intake ratio; PR = preference ratio. The beagle dogs were fed 0% (Con), 0.25% (Low_Gal), 0.5% (Mid_Gal), and 1% (High_Gal) L-β-galactoglucan.

**Table 3:** Effect of L-β-galactoglucan supplementation on body weight change and fecal and hair quality in the dogs.

Items	Con	Low_Gal	Mid_Gal	High_Gal	SEM	*p*-Value
Fecal score	2.63	2.68	2.73	2.65	0.019	0.209
IBW (kg)	13.4	13.8	12.1	13.7	0.387	0.381
FBW (kg)	13.7	13.3	11.5	13.3	0.406	0.247
Weight change (kg)	0.35 ^a^	−0.51 ^b^	−0.55 ^b^	−0.56 ^b^	0.098	<0.01
Hair score	6.43	7.23	7.55	6.73	0.194	0.166

Data are presented as mean ± Standard Error of the Mean. ^a,b^ means with different superscript letters were significantly different (*p* < 0.05). IBW = initial body weight; FBW = final body weight. The beagle dogs were fed 0% (Con), 0.25% (Low_Gal), 0.5% (Mid_Gal), and 1% (High_Gal) L-β-galactoglucan.

**Table 4:** Effect of L-β-galactoglucan supplementation on the apparent total tract digestibility of nutrients in the dogs.

Items	Con	Low_Gal	Mid_Gal	High_Gal	SEM	*p*-Value
CP (%)	95.5	95.6	95.5	95.1	0.167	0.738
EE (%)	95.2	95.5	95.3	95.7	0.144	0.698
OM (%)	95.10	94.98	95.41	94.96	0.117	0.516
CF (%)	71.3	70.1	72.4	71.1	1.08	0.921

Data are presented as mean ± Standard Error of the Mean. CP = crude protein; EE = ether extract; OM = organic matter; CF = crude fiber. The beagle dogs were fed 0% (Con), 0.25% (Low_Gal), 0.5% (Mid_Gal), and 1% (High_Gal) L-β-galactoglucan.

**Table 5:** Effect of L-β-galactoglucan supplementation on the hematology of the dogs.

Items	Con	Low_Gal	Mid_Gal	High_Gal	SEM	*p*-Value
WBC (10^9^/L)	11.4	11.4	11.67	12.0	0.47	0.967
RBC (10^12^/L)	7.17	7.11	7.24	7.17	0.14	0.992
HGB (g/L)	163	158	157	154	3.65	0.841
PLT (10^9^/L)	275	293	342	295	12.95	0.312
HCT (%)	47.7	46.1	47.4	46.9	0.92	0.944
MCV (fL)	64.8	63.6	65.5	65.3	0.34	0.216
MCH (pg)	21.7	21.5	21.6	21.5	0.09	0.869

Data are presented as mean ± Standard Error of the Mean. WBC = white blood cell; RBC = red blood cell; HGB = hemoglobin; PLT = platelet; HCT = hematocrit; MCV = mean cell volume; MCH = mean corpuscular hemoglobin. The beagle dogs were fed 0% (Con), 0.25% (Low_Gal), 0.5% (Mid_Gal), and 1% (High_Gal) L-β-galactoglucan.

**Table 6:** Effect of L-β-galactoglucan supplementation on the serum biochemistry parameters of the dogs.

Items	Con	Low_Gal	Mid_Gal	High_Gal	SEM	*p*-Value
Glu (mmol/L)	5.64	5.57	6.07	5.98	0.093	0.142
TP (g/L)	59.1	59.7	61.9	61.2	0.614	0.352
ALB (g/L)	32.8	32.2	31.8	31.4	0.346	0.616
GLB (g/L)	26.4	27.6	30.1	29.9	0.573	0.052
ALT (U/L)	38.6	40.6	50.5	33.5	3.815	0.474
AST (U/L)	32.7	37.7	34.9	27.7	1.386	0.072
TG (mg/dL)	60.5	56.2	50.0	42.5	3.211	0.06
HDL-C (mg/dL)	150	124	128	136	4.417	0.157
LDL-C (mg/dL)	9.75	9.45	6.20	8.96	0.540	0.069

Data are presented as mean ± Standard Error of the Mean. GLU = glucose; TP = total protein; ALB = albumin; GLB = globulin; AST = aspartate aminotransferase; ALT = alanine aminotransferase; TG = triglyceride; HDL-C = high-density lipoprotein cholesterol; LDL-C = low-density lipoprotein cholesterol. The beagle dogs were fed 0% (Con), 0.25% (Low_Gal), 0.5% (Mid_Gal), and 1% (High_Gal) L-β-galactoglucan.

**Table 7:** Effect of L-β-galactoglucan supplementation on the serum immunoglobulin of the dogs.

Items	Con	Low_Gal	Mid_Gal	High_Gal	SEM	*p*-Value
IgA (μg/mL)	1415	1402	1463	1448	0.017	0.578
IgG (μg/mL)	1148	1112	1131	1113	0.007	0.195
IgM (μg/mL)	49.6	48.2	46.2	47.4	0.569	0.189

Data are presented as mean ± Standard Error of the Mean. IgA = immunoglobulin A; IgM = immunoglobulin M; IgG = immunoglobulin G. The beagle dogs were fed 0% (Con), 0.25% (Low_Gal), 0.5% (Mid_Gal), and 1% (High_Gal) L-β-galactoglucan.

## Data Availability

All data generated or analyzed during this study are available from the corresponding author upon request.
